# Autoimmunity/inflammation in a monogenic primary immunodeficiency cohort

**DOI:** 10.1038/cti.2017.38

**Published:** 2017-09-15

**Authors:** William Rae, Daniel Ward, Christopher J Mattocks, Yifang Gao, Reuben J Pengelly, Sanjay V Patel, Sarah Ennis, Saul N Faust, Anthony P Williams

**Affiliations:** 1 Department of Immunology, University Hospital Southampton NHS Foundation Trust, Southampton, UK; 2 Southampton NIHR Wellcome Trust Clinical Research Facility, University of Southampton, University Hospital Southampton, Southampton, UK; 3 Wessex Regional Genetics Laboratory, Salisbury District Hospital, Salisbury, UK; 4 Wessex Investigational Sciences Hub Laboratory, University of Southampton, University Hospital Southampton NHS Foundation Trust, Southampton, UK; 5 NIHR Cancer Research UK Experimental Cancer Medicine Centre, Southampton, UK; 6 Human Genetics and Genomic Medicine, Faculty of Medicine, University of Southampton, Southampton, UK; 7 Department of Paediatric Immunology and Infectious Diseases, University Hospital Southampton NHS Foundation Trust, Southampton, UK; 8 Faculty of Medicine, University of Southampton, Southampton, UK; 9 Institute for Life Sciences, University of Southampton, Southampton, UK

## Abstract

Primary immunodeficiencies (PIDs) are rare inborn errors of immunity that have a heterogeneous phenotype that can include severe susceptibility to life-threatening infections from multiple pathogens, unique sensitivity to a single pathogen, autoimmune/inflammatory (AI/I) disease, allergies and/or malignancy. We present a diverse cohort of monogenic PID patients with and without AI/I diseases who underwent clinical, genetic and immunological phenotyping. Novel pathogenic variants were identified in *IKBKG*, *CTLA4*, *NFKB1*, *GATA2*, *CD40LG* and *TAZ* as well as previously reported pathogenic variants in *STAT3*, *PIK3CD*, *STAT1*, *NFKB2* and *STXBP2*. AI/I manifestations were frequently encountered in PIDs, including at presentation. Autoimmunity/inflammation was multisystem in those effected, and regulatory T cell (Treg) percentages were significantly decreased compared with those without AI/I manifestations. Prednisolone was used as the first-line immunosuppressive agent in all cases, however steroid monotherapy failed long-term control of autoimmunity/inflammation in the majority of cases and additional immunosuppression was required. Patients with multisystem autoimmunity/inflammation should be investigated for an underlying PID, and in those with PID early assessment of Tregs may help to assess the risk of autoimmunity/inflammation.

Primary immunodeficiencies (PIDs) encompass a collection of rare inborn errors of immunity often with broad overlapping phenotypes that include severe susceptibility to life-threatening infections from multiple pathogens, unique sensitivity to a single pathogen, autoimmune/inflammatory (AI/I) disease, allergies and/or malignancy.^1^ Over 300 monogenic causes for PIDs have now been identified, which has increased the diversity of clinical phenotypes that is encountered in clinical practice.^2^

Advances in the treatment and prophylaxis of infection have improved the quality of life and prognosis for patients with PID. Treatments such as immunoglobulin (Ig) replacement and antimicrobial agents are now highly effective at preventing and treating infections in many PIDs. However, with the improved management of infection, AI/I are becoming an increasing cause of morbidity and mortality.^3^ AI/I manifestations are frequently observed in PIDs due to inherent impairment of regulatory functions within the immune system.^4, 5^ Failure to maintain self-tolerance results in self-epitope-specific adaptive immune responses and autoimmunity, and failure to regulate innate immune responses results in autoinflammation in the absence of detectable self-reactive adaptive immune responses. Many PID conditions impair one or more immunological components required for immune system regulation, and AI/I manifestations are prevalent in PID cohorts across a range of monogenic PIDs.^3^

To investigate the varied presentation and frequency of AI/I diseases in PID we recruited a cohort of monogenic PID patients as classified within the 2015 International Union of Immunological Societies.^2^ We evaluated the prevalence of AI/I manifestations in this cohort, and investigated whether any immunological, genetic or phenotypic features correlated with the development of AI/I. We also describe the treatments and outcomes for the AI/I manifestations across the cohort.

## Results

### Genetic investigations

A phenotypically heterogeneous cohort of 16 participants with monogenic PID was recruited from a single PID centre (Supplementary Information: Clinical phenotypes). Participants underwent either whole-exome sequencing, an extended PID gene panel or targeted single-gene sequencing. Novel pathogenic variants were identified in *IKBKG*, *CTLA4*, *NFKB1*, *GATA2*, *CD40LG* and *TAZ*. Previously reported pathogenic variants were identified in *STAT3*, *PIK3CD*, *STAT1*, *NFKB2* and *STXBP2* (Table 1).

### AI/I manifestations

The initial clinical presentati1on was due to infection in 62% (10/16) of cases and AI/I disease in 38% (6/16) of cases. During follow-up, a further 3 participants developed AI/I manifestations, resulting in a total 56% (9/16) of the participants in the cohort experiencing AI/I disease that required medical intervention. Autoimmune cytopenias were the most frequently encountered AI/I complication (*n*=7). Other organ-specific AI/I manifestations effected the gastrointestinal (GI; *n*=4), pulmonary (*n*=3), hepatic (*n*=2), cutaneous (*n*=2) and renal (*n*=1) organ systems (Table 1). AI/I disease was multisystem in all effected participants.

### T-cell subsets in participants with and without autoimmunity/inflammation

**Figure 1:**
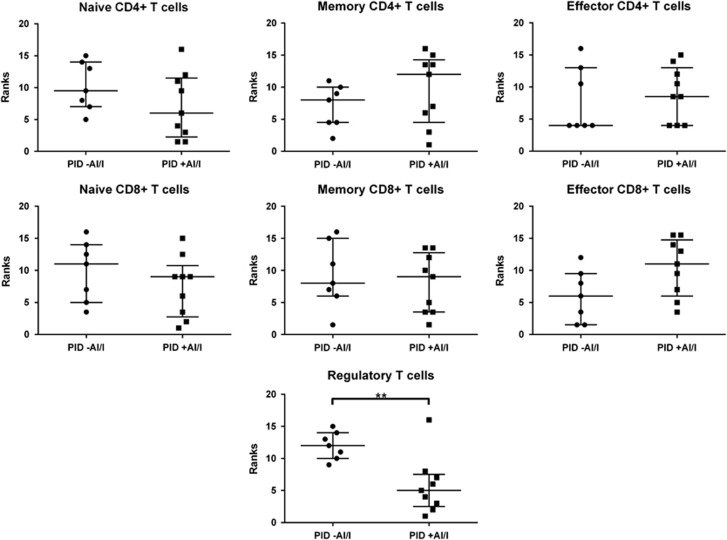
T-cell subgroups compared between the groups, PID without AI/I (PID −AI/I) and PID with AI/I (PID +AI/I) (median and interquartile range). Tregs were significantly reduced in PID +AI/I compared with PID −AI/I (*P*=0.0079). *n*=2. *P<0.01.

Participants were grouped into those without AI/I (PID −AI/I) and those with AI/I (PID +AI/I) (Supplementary Table 1). Analysis of peripheral naive T cells (defined as CD3^+^ CD4^+^ or CD8^+^, CD27^+^ and CD45RA^+^), memory T cells (defined as CD3^+^, CD4^+^ or CD8^+^, CD27^+/−^ and CD45RA^−^) and effector T cells (defined as CD3^+^, CD4^+^ or CD8^+^, CD27^−^ and CD45RA^+^) was performed (Supplementary Figure 1).^6, 7^ Analysis of regulatory T cells (Tregs) (defined as CD3^+^, CD4^+^, CD25^+^ and CD127^low^) was also performed (Supplementary Figure 2). Treg percentages were significantly decreased in the PID +AI/I group compared with PID −AI/I (*P*=0.0079; Figure 1). The PID +AI/I group showed a trend towards increased effector CD8^+^ cells (Figure 1; Supplementary Table 2) but results were not statistically significant compared with the PID −AI/I group. Other T-cell subsets were not significantly different between the groups (Figure 1; Supplementary Table 2).

### Treatment interventions for autoimmunity/inflammation

**Figure 2:**
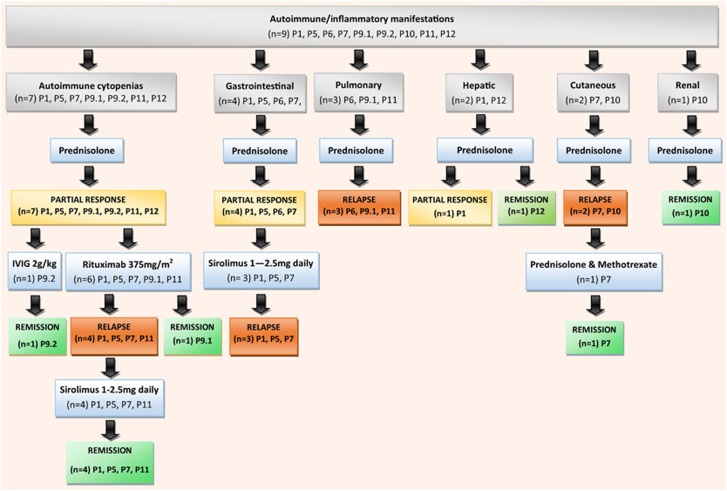
Diagram illustrating the treatments for AI/I manifestations within the cohort. Participants had multisystem AI/I and often treatments were only efficacious for a single AI/I manifestation in individuals. Prednisolone monotherapy appeared ineffective for the majority of AI/I conditions encountered in PID. Remission, complete normalisation of laboratory parameters and/or clinical symptoms; partial response, improvement to near normal and stabilisation in laboratory parameters and/or clinical symptoms; relapse, no improvement/continue deterioration in laboratory parameters and/or clinical symptoms.

Treatment inventions for AI/I manifestations were initiated based on clinical disease and symptoms. Prednisolone was used as first-line immunosuppression in all participants with AI/I (*n*=9; Figure 2). Autoimmune cytopenias occurred in 7/16 participants (Table 1), and prednisolone 1 mg kg^−1^ per day resulted in an initial clinical response in 7/7 participants. All 7/7 participants subsequently required additional immunomodulation due to refractory/relapsed autoimmune cytopenias during prednisolone weaning. As second-line treatment for autoimmune cytopenias, 6/7 relapsed participants received rituximab and 1/7 was given Ig 2 g kg^−1^. Of the 6 participants who required rituximab, 4/6 needed a further long-term steroid sparing agent due to recurrence of autoimmune cytopenias post rituximab. Sirolimus (1–2.5 mg per day) was the most effective steroid sparing at maintaining remission for autoimmune cytopenias in 4/4 participants.

GI AI/I manifestations partially responded to prednisolone in 4/4 participants. On weaning prednisolone GI disease returned and sirolimus did not adequately control GI AI/I in all 3/3 participants. Pulmonary disease was not controlled by prednisolone monotherapy in any of the participants, and radiological and lung function continue to decline. Liver AI/I responded to prednisolone in 2/2 participants, but relapsed shortly after withdrawal in 1/2 participants.

It was observed that a specific immunosuppressive therapy often improved one organ-specific AI/I complication in an individual, but failed to effectively treat other multisystem AI/I disease in the same individual. Examples of this include that a slow weaning course of prednisolone achieved complete long-term remission of the renal tubular acidosis in P10, but did not cause any clinical response in the alopecia areata. Similarly, in P7, there was a deterioration in cutaneous and GI AI/I disease whilst on sirolimus monotherapy, despite remission of autoimmune cytopenias. This mixed response necessitated an alteration in treatment to prednisolone 1 mg kg^−1^ per day in combination with methotrexate (7.5 mg per week), which resolved the cutaneous AI/I (Figure 2).

## Discussion

As the list of PIDs grows so does the number of AI/I manifestations reported.^2, 8^ AI/I disease may be the major presenting symptom for a significant proportion of PID patients. As may be expected, the prevalence of AI/I disease appears to increase with age in PID cohorts and effects a significant proportion of patients.^3^ The pathophysiology that gives rise to AI/I in PIDs is varied and proposed mechanisms include; absolute lymphopenia causing a lack of regulatory lymphocytes, apoptosis defects preventing removal of self-reactive adaptive immune responses, over-activation and dysregulation of lymphocytes, defects of central tolerance, increased and unregulated type 1 interferon responses, and complement defects impairing the removal of immune complexes and cell debris.^4^

Autoimmune cytopenias are a common AI/I manifestation encountered across PIDs, and reports suggest that PID is subsequently diagnosed in up to 50% of paediatric cases of refractory multi-lineage autoimmune cytopenia (Evans syndrome).^9, 10^ This high prevalence of autoimmune cytopenias in PID was also apparent within our cohort with 7/16 of participants developing autoimmune cytopenia of one or more cell lineages (Table 1). Therefore ‘difficult-to-treat’ Evans syndrome may indicate an underlying PID and is a frequent AI/I in clinical care.

AI/I diseases can affect all subgroup classifications of PID, but is more frequently encountered in T-cell defects and predominantly antibody defects, particularly common variable immunodeficiency.^1, 3^ Our cohort demonstrates similar characteristics with 4/5 participants with predominantly antibody deficiencies suffering AI/I (Table 1). In those with inherent T-cell defects (mutations in genes that are significantly expressed in T cells: *IKBKG*; *STAT3*; *CTLA4*; *STAT1*; *STXBP2*; *CD40LG*; and *TAZ*^11^) a significant proportion (4/10) also suffered AI/I (Table 1).

The broad genetic pleiotropy of PID patients covers a diverse array of AI/I manifestations. Previous cohort and case reports describe AI/I disease observed in cases of monogenic PIDs, and we outline the similarities and differences of previous reports compared with our participants phenotypes (Supplementary Information: Clinical phenotypes).

### *IKBKG* (*NEMO*) deficiency (OMIM 300291)

P1 (*IKBKG* p.R63Q) suffered with Evans syndrome, colitis and granulomatous hepatitis. Autoimmune haemolytic anaemia and immune thrombocytopenia have both been reported in *IKBKG* deficiency, and colitis is a common inflammatory complication.^12, 13, 14^ Hepatic granuloma have been only been reported in hypofunctional *IKBKG* due to disseminated mycobacterial infection.^13^ A liver biopsy performed on P1 found no evidence of mycobacteria or other pathogens, suggesting that the granuloma are sterile and due to immune dysregulation. Larger studies of *IKBKG* deficiency patients will help to expand the reported phenotype in this condition.

### *STAT3* dominant negative hyper IgE syndrome (OMIM 147060)

P2 (*STAT3* p.G618D) and P3 (*STAT3* p.V637M), both with hyper IgE syndrome due to loss-of-function variants in *STAT3* and did not demonstrate any AI/I manifestations.^15^ Non-infectious complications are common in hyper IgE syndrome, as was the case in our participants (Supplementary Information: Clinical phenotypes) but these are not believed to have an AI/I pathophysiology. In contrast, *STAT3* GOF variants present with a phenotype of multisystem AI/I, which may support that *STAT3* LOF patients are relatively protected from AI/I.^16, 17^

### *PIK3CD* activated PI3K delta syndrome (OMIM 615513)

P4 and P5 (both *PIK3CD* p.E1021K) showed discordance for autoimmune diseases, with P4 having no AI/I disease and P5 suffering from AIHA and lymphocytic colitis. AI/I disease is frequent in *PIK3CD* GOF patients with 42% of patients having some form of AI/I in reported cohorts.^18^

### *CTLA4* insufficiency (OMIM 616100)

P6 (*CTLA4* p.A54T) and P7 (*CTLA4* p.V40M) both suffered with multisystem AI/I.^19, 20^ The clinical phenotype of *CTLA4* insufficiency is heterogeneous with a wide range of organ-specific AI/I being described in the disease. Enteropathy is reported in up to 78% of cases and was present in both P6 and P7.^19^ Interstitial lung disease was also present in P6 and is reported in 66% of *CTLA4* cases.^19^ Autoimmune haemolytic anaemia and immune thrombocytopenia are also commonly encountered at 28% and 35% of cases, respectively, and psoriasis 21% of cases,^19^ all of which were also present in P7.

### *STAT1* gain of function (OMIM 614162)

P8.1 and P8.2 (*STAT1* p.R274Q GOF) did not develop any AI/I disease during follow-up. A large *STAT1* GOF cohort reported AI/I in 37% of patients, with a slight preponderance in female patients.^21^ Thyroid disease was the most common AI/I reported (22%), but skin disease (10%) and autoimmune cytopenias (4%) were also frequently reported. Further reports have further broadened the phenotype of *STAT1* GOF to include ‘IPEX-like’ presentations with multisystem AI/I.^22^ The janus kinase inhibitor ruxolitinib has shown promise in targeted AI/I in *STAT1* GOF patients as a targeted immunosuppressive, as well as having benefits on chronic mucocandidasis.^23^

### *NFKB1* haploinsufficiency (OMIM 616576)

P9.1 and P9.2 (*NFKB1* p.S302Ffs*7) both suffered autoimmunehaemolytic anaemia, which is reported in *NFKB1* haploinsufficient patients.^24, 25^ Differing AI/I is observed in patients with *NKFB1* mutations, ranging from antibody deficiency, Behcet-like disease, to an autoinflammatory phenotype.^26^

### *NFKB2* dominant negative immunodeficiency (OMIM 615577)

P10 (*NFKB2* p.R853*) suffered autoimmune alopecia, which is widely reported in patients with dominant negative *NFKB2* variants but the renal disease that was present in P10 has not been reported in *NKFB2* variants to date.^27, 28^ The pituitary adrenal axis is often effected in *NFKB2*, but was normal in P10, although this is not believed to be an AI/I phenomenon; instead due to hypoplasia of the anterior pituitary.^27, 28, 29^ Further large-scale studies are needed to catalogue the frequencies and phenotype of AI/I in *NFKB1* and *NFKB2* patients.

### *GATA2* haploinsufficiency (OMIM 614172)

*GATA2* haploinsufficiency is described as protean disorder that may present with a variety of clinical phenotypes.^30^ Phenotypes include dendritic cell, monocyte, B and natural killer cell deficiency with mycobacterial infections (MonoMAC), myelodysplastic syndromes, acute myeloid leukaemia and Emberger syndrome. Viral and mycobacterial infections are the most commonly encountered pathogens in *GATA2* haploinsufficiency.^30^ *GATA2* deficiency usually causes cytopenias due to impaired bone marrow haematopoiesis and myelodysplasia, but the elevated levels of autoreactive peripheral CD38^−^ CD21^−^ B cells described in the periphery of *GATA2* patients may increase the risk of antibody-mediated autoimmunity,^31^ and P11 (*GATA2* p.T176P) suffered with recurrent Evans syndrome. Lung involvement with alveolar proteinosis occurs in *GATA2* haploinsufficient patients due to impairment of alveolar macrophages, but lung fibrosis has also been reported recently and was observed in P11.^32, 33^

### *STXBP2* deficiency (OMIM 613101)

P12 (*STXBP2* c.1247-1 homozygous) developed autoimmune neutropenia primary sclerosing cholangitis with dysgammaglobulinaemia, after initially presenting with haemophagocytic lymphohistiocytosis (Supplementary Information: Clinical phenotypes). Presentations of individuals with the same homozygous *STXBP2* variant 1247-1G>C have also been described with dysgammaglobulinaemia and autoimmune liver involvement in the absence of haemophagocytic lymphohistiocytosis.^34, 35^

### *CD40LG* deficiency (OMIM 308230)

P13 (*CD40LG* p.A141P) presented with raised IgM, absent IgG and IgA, and necrotic pseudomonal tonsillitis. Stimulated CD4^+^ T cells showed absent expression of CD40L on the cell surface. *CD40LG*-deficient patients frequently develop autoimmunity, however P13 has no evidence of AI/I disease to date. At odds with reports of reduced Treg frequency in CD40LG patients, P13 has raised Tregs at 15.4% (Supplementary Table 3), which may be relatively protective against AI/I development in this case.^36^

### *TAZ* deficiency (OMIM 302060)

P14 (*TAZ* p.K220E) has significant T-cell lymphopenia, which is one aetiology believed to predispose to AI/I disease in PID.^4^ The intrinsic apoptosis pathway is also defective in Barth syndrome due to impairment of mitochondria initiation of apoptosis.^37^ Despite these potential mechanistic risks for AI/I development,^4^ AI/I are not widely reported in Barth syndrome patients. Recently *TAZ* has been described to regulate Th17 and Treg development, and *TAZ*-deficient lymphocytes show impaired Th17 and increased Treg differentiation.^38^ This lymphocyte defect may protect Barth syndrome patients from AI/I disease.

These previous reports and comparisons with our cohort illustrate the prevalence and heterogeneity of AI/I that is encountered in the clinical care of patients with PID. It is also apparent that multisystem AI/I is frequent in PID, and that patients presenting with complex multisytem AI/I should be investigated for PID.

The need to identify markers of impending AI/I in PID has long been recognised.^39^ Tregs appeared reduced across our cohort of PID with AI/I, and may present a potential indicator for the risk of developing AI/I in patients. However, further work is required with larger studies to confirm these findings. Because of the heterogeneity of PID there are also limitations of this approach when applied to individual cases, such as raised Treg percentages with impaired function in cases of *CTLA4*-insufficient patients with AI/I.

Decisions on treatment options for AI/I in PIDs are challenging due to the inherent risks of iatrogenic immunosuppression in immunocompromised individuals. Multisystem AI/I poses further challenges, as one AI/I manifestation may respond to a therapy, whereas another can remain refractory to the same therapy. It is hoped that ‘precision medicines’ targeted to the underlying genetic abnormality will provide a more holistic therapeutic option for multisystem AI/I.^10, 40, 41^ Currently, due to the rarity of individual monogenic PIDs, there is a relative lack of large-scale studies of these precision treatments, and financial limitations within health-care systems still limit the wide-scale adoption of precision medicine at the bedside.

Our experience of a heterogeneous cohort of PID patients suggests that for autoimmune cytopenias, first-line prednisolone, second-line rituximab and third-line sirolimus is an effective treatment regime. This is a similar treatment pathway to that described for Evans syndrome in non-PID patients, autoimmune lymphoproliferative syndrome^42, 43^ and common variable immunodeficiency,^44^ indicating that this regime can be extrapolated across PIDs with autoimmune cytopenia. Several guidelines for the treatment of autoimmune cytopenias include mycophenolate mofetil as the second-line agent within treatment algorithms.^45, 46^ Whilst mycophenolate is often including in treatment pathways, our experience of severe autoimmune cytopenias in PID is that sirolimus appears more efficacious in difficult-to-treat cytopenias associated with PID. Prednisolone monotherapy appears ineffective at long-term control of AI/I conditions in PID. Organ-specific AI/I disease in PID often requires additional immunosuppression, such as rituximab and mycophenolate in pulmonary disease to produce a clinical benefit.^47, 48^ Therefore, when considering therapeutic immunosuppression it appears that the site/tissue effected by AI/I should influence treatment choices.

In conclusion multisystem AI/I manifestations are frequently encountered across a range of monogenic PIDs in clinical care. Multisystem AI/I present in PID makes treatment options challenging, and steroid monotherapy appears ineffective in the longer term for many AI/I diseases in PID. There still remains a need to develop methods of pre-empting AI/I in PID, and although Tregs were reduced in those with AI/I there are caveats to this and further studies are needed to confirm these findings.

## Methods

### Human samples

Whole-blood EDTA and lithium heparinised samples were collected from controls and patients with PID at a single centre. All participants with PID had monogenic diagnoses of PID listed in the International Union of Immunological Societies classification.^2^ Informed consent was obtained from all participants included in the study. All studies were approved by the institutional review board (Research Ethics Committee reference 12/NW/0794).

### Lymphocyte phenotyping

Whole-blood lymphocyte immunophenotyping was performed by flow cytometry on a FACS Canto II (BD Biosciences, San Jose, CA, USA). T-, B- and natural killer cell phenotyping was performed using CD45-PerCP-Cy5.5 (clone 2D1), CD3-FITC (clone SK7), CD4-PE-Cy7 (clone SK3), CD8-APC-Cy7 (clone SK1), CD19-APC (clone SJ25C1), CD16-PE (clone B73.1) and CD56-PE (clone NCAM 16.2). T-cell memory phenotyping: CD3-PerCP-Cy5.5 (clone 2D1); CD4-PE-Cy7 (clone SK3); CD8-APC (clone SK1); CD27-PE (clone L128); and CD45RA-FITC (clone L48). B-cell memory phenotyping: CD19-FITC (clone SJ25C1); CD27-APC (clone L128); and IgM-PE (clone SA-DA4, Beckman Coulter, Los Angeles, CA, USA). αβ and γδ T cells were assessed using CD3-PerCP-Cy5.5 (clone 2D1), αβ TCR-FITC (clone WT31) and γδ TCR-PE (clone 11F2). Tregs were phenotyped with CD3-PerCP-Cy5.5 (clone 2D1), CD4-APC (clone SK3), CD25-PE (clone 2A3) and CD127-BV450 (clone HIL-7R-M21) (all BD Biosciences). Flow cytometry plots for naive (CD3^+^, CD4^+^ or CD8^+^, CD27^+^ and CD45RA^+^), memory (CD3^+^, CD4^+^ or CD8^+^, CD27^−/+^ and CD45RA^−^), effector (CD3^+^, CD4^+^ or CD8^+^, CD27^−^ and CD45RA^+^) T cells and Tregs (CD3^+^, CD4^+^, CD25^+^ and CD127^low^) were analysed using FlowJo (LLC, Ashland, OR, USA).

### Igs and antibody responses

IgG, IgA, IgM and IgE were assessed by nephelometry according to the manufacturer’s instructions (Beckman Coulter). Pneumococcal and tetanus IgG responses were assessed by commercial enzyme-linked immunosorbent assay according to the manufacturer’s instructions (Binding Site, Birmingham, UK).

### Genetic analysis

DNA was extracted from EDTA blood samples using QIAamp DNA blood mini kit (Qiagen, Hilden, Germany) according to the manufacturer’s instructions. DNA quality was checked by Nanodrop spectrometry (Thermofisher, Waltham, MA, USA). Genetic analysis was performed by whole-exome sequencing (P5, P8.1, P8.2 and P10) using the TruSight One panel kit (Illumina, San Diego, CA, USA; P1, P4, P6, P7, P9, P11 and P12) and by single-candidate gene analysis (P2 and P3). Data were processed according to GATK best practice guidelines and aligned to GRCh37/hg19 reference genome. Variants identified in this study have been submitted to ClinVar NCBI.

Variant interrogation was performed using in silico predictive tools Polyphen2,^49^ SIFT^50^ and Exome Aggregation Consortium,^51^ supported by Sapientia (Congenica, Cambridge, UK) and Ensembl.^52^ Variants pathogenicity was grading according to the American College of Medical Genetics criteria.^53^

### Participant grouping

Participants were grouped into those with PID and AI/I manifestations (PID +AI/I) and those without AI/I (PID −AI/I). Both groups had similar characteristics, including mean age (Supplementary Table 1).

### Clinical responses

Clinical responses were graded similarly to previous studies.^54^ Remission=complete normalisation of laboratory parameters and/or complete resolution of clinical symptoms. Partial response=improvement to near normal laboratory parameters with stabilisation of results and/or improvement in clinical manifestations (for example, reduction in diarrhoea frequency). Relapse=little or no improvement in laboratory parameters and/or no improvement in clinical symptoms (for example, diarrhoea frequency and skin inflammation) and/or no improvement/progressive deterioration in imaging (for example, increased infiltrates in lungs and reducing lung function).

### Statistical analysis

Because of skewed distributions of T-cell subsets (Supplementary Figure 3), unpaired Mann–Whitney *U*-test was used for analysis (GraphPad Software, La Jolla, CA, USA). *P*<0.05 was used as significance cutoff. Graphs display Mann–Whitney *U* Ranks (Figure 1). Data distribution was calculated using SPSS v27 (IBM, Armonk, NY, USA; Supplementary Figure 3). Figures were created using Prism: GraphPad.

## Figures and Tables

**Table 1:** Genetic, infection and AI/I characteristics in the PID cohort

	*Genetic variant*	*ACMG variant interpretation*	*IUIS main PID category*	*Infections*	*Autoimmunity/inflammation*	*Immunomodulatory treatment*
P1	*IKBKG* c.185G>A:p.(Arg62Gln)	Likely pathogenic (IV) (PM2, PM5, PM6, PP2, PP3)	Combined immunodeficiency with associated features	*H. influenzae*, Norovirus	AIHA, ITP, lymphocytic colitis, granulomatous hepatitis	Prednisolone, rituximab, sirolimus
						
P2	*STAT3*_LOF_ c.1853G>A:p.(Gly618Asp)	Pathogenic (II) (PS1, PS3, PM2, PP2)	Combined immunodeficiency with associated features	*Pneumocystis jivorecii*, *S. aureus*, *S.* *pneumoniae*, *H. influenzae*		
						
P3	*STAT3*_LOF_ c.1909G>A:p.(Val637Met)	Pathogenic (II) PS1, PS3, PS4, PM2, PP1-M)	Combined immunodeficiency with associated features	*Aspergillus fumigates*, *S. aureus*, *H. influenzae*		
						
P4	*PIK3CD*_GOF_ c.3061G>A:p.(Glu1021Lys)	Pathogenic (II) (PS1, PS3, PS4, PM1, PM2, PM6)	Predominantly antibody deficiencies	Chronic mucocutaneous candidasis, *H. influenzae*		
						
P5	*PIK3CD*_*GOF*_ c.3061G>A:p.(Glu1021Lys)	Pathogenic (II) (PS1, PS3, PS4, PM2, PM1)	Predominantly antibody deficiencies	*S. pneumoniae*	AIHA, lymphocytic colitis	Prednisolone, rituximab, mycophenolate, sirolimus
						
P6	*CTLA4* c.160G>A:p.(Ala54Thr).	Likely pathogenic (V) (PM2, PM6, PPS, PP3, PP4)	Diseases of immune dysregulation	*H. influenzae*, *H. parainfluenzae*, *Pseudomonas aeruginosa*, *Clostridium difficile*	Pulmonary fibrosis, lymphocytic colitis	Prednisolone
						
P7	*CTLA4* c.118G>A:p.(Val40Met)	Likely pathogenic (IV) (PM2, PM5, PM6, PP3)	Diseases of immune dysregulation	*S. pneumoniae, Influenza H1N1*, *Candida krusei*, *S. aureus*, CMV	AIHA, ITP, autoimmune neutropenia, psoriasis, lymphocytic colitis	Prednisolone, rituximab, ciclosporin, sirolimus, methotrexate
						
P8.1	*STAT1*_GOF_ c.821G>A:p.(Arg274Gln)	Pathogenic (II) (PS1, PS3, PS4, PP1-S, PM2, PP1-M)	Defects of innate and intrinsic immunity	Chronic mucocutaneous candidasis, *S. aureus*, *Pseudomonas aeruginosa*, *H. influenzae*		
						
P8.2	*STAT1*_GOF_ c.821G>A:p.(Arg274Gln)	Pathogenic (II) (PS1, PS3, PS4, PP1-S, PM2, PP1-M)	Defects of innate and intrinsic immunity	Chronic mucocutaneous candidasis		
						
P9.1	*NFKB1* c.904dupT:p.(Ser302Phefs*7)	Pathogenic (Ia) (PVS1, PM6, PP1-S, PP3)	Predominantly antibody deficiencies	*H. influenzae*	AIHA, pulmonary fibrosis	Prednisolone, rituximab
						
						
P9.2	*NFKB1* c.904dupT:p.(Ser302Phefs*7)	Pathogenic (Ia) (PVS1, PM6, PP1-S, PP3)	Predominantly antibody deficiencies		AIHA, ITP, autoimmune neutropenia	Prednisolone, immunoglobulin 2 g kg^−1^
P10	*NFKB2* c.2557C>T:p.(Arg853*)	Pathogenic (Ia) (PVS1, PS1, PS3, PP1-S, PM2)	Predominantly antibody deficiencies	*H. parainfluenzae*, *H. influenzae*, *M. catarrhalis*, *S. aureus*, adenovirus	Renal tubular acidosis, alopecia areata	Prednisolone
						
						
P11	*GATA2* c.526A>C:p.(Thr176Pro)	Likely pathogenic (IV) (PM2, PM6, PP3, PP1-S, PM2)	Congenital defects of phagocyte number, function or both	*H. influenza*, *Mycoplasma pneumoniae*	AIHA, ITP, pulmonary fibrosis	Prednisolone, rituximab, sirolimus
						
P12	*STXBP2* c.1247-1G>C homozygous	Pathogenic (Ia) (PVS1, PS1, PS3, PM2, PM3, PP3, PP4)	Diseases of immune dysregulation	EBV, HSV1	Autoimmune neutropenia, autoimmune sclerosing cholangitis	Prednisolone, rituximab
P13	*CD40LG* c.421C>G:p.(Ala141Pro)	Pathogenic (II) (PP1-S, PS3, PS4, PM2, PP1)	Immunodeficiencies affecting cellular and humoral immunity	*Pseudomonas aeruginosa*		
						
P14	*TAZ* c.658A>G:p.(Lys220Glu)	Likely pathogenic (V) (PM2, PM6, PP3, PP4)	Congenital defects of phagocyte number, function or both	*S. pneumoniae*, *S. agalactiae*, *N. meningitidis*		

Abbreviations: ACMG, American College of Medical Genetics; AIHA, Autoimmune haemolytic anaemia; AI/I, autoimmune/inflammatory; CMV, Cytomegalovirus; EBV, Epstein-Barr virus; *H. influenzae*, *Haemophilus influenzae*; *H. parainfluenzae*, *Haemophilus parainfluenzae*; HSV1, Herpes simplex virus-1; ITP, immune thrombocytopenia; IUIS, International Union of Immunological Societies; *M. catarrhalis*, *Moraxella catarrhalis*; *N. meningitidis*, *Neisseria meningitidis*; PID, primary immunodeficiency; *S. agalactiae*, *Streptococcus agalactiae*; *S. aureus*, *Staphylococcus aureus*; *S. pneumoniae*, *Streptococcus pneumoniae*.
