# Supplementary material for: Autoimmunity/inflammation in a monogenic primary immunodeficiency cohort
Source: Clin Transl Immunology. 2017 Sep 15;6(9):e155–. doi: 10.1038/cti.2017.38 (PMC5628267; doi:10.1038/cti.2017.38)
Supplement: Supplementary Information [file cti201738x1.docx]

**Supplementary Information**

**Autoimmunity/inflammation in a monogenic primary immunodeficiency cohort**

William Rae^1, 2^, Daniel Ward^3^, Christopher J. Mattocks^3^, Yifang Gao^3, 4^ Reuben J. Pengelly^5^, Efrem Eren^1^, Sanjay V. Patel^6^, Sarah Ennis^5^, Saul N. Faust^2, 6, 7^, Anthony P. Williams^1, 3, 4^.

^1^ Department of Immunology, University Hospital Southampton NHS Foundation Trust, UK. ^2^ Southampton NIHR Wellcome Trust Clinical Research Facility, University of Southampton, University Hospital Southampton, Southampton, UK. ^3^ Wessex Investigational Sciences Hub Laboratory, University of Southampton, University Hospital Southampton NHS Foundation Trust, UK. ^4^ NIHR Cancer Research UK Experimental Cancer Medicine Centre, Southampton, UK. ^5^Human Genetics and Genomic Medicine, Faculty of Medicine, University of Southampton, Southampton, UK. ^6^ Department of Paediatric Infectious Diseases and Immunology, Southampton Children's Hospital, UK. ^7^ Faculty of Medicine, University of Southampton, UK

Corresponding author;

William Rae, Department of Immunology, University Hospital Southampton NHS Foundation Trust, UK. will.rae@nhs.net

**Table of Contents**

[Clinical phenotypes 2](#_Toc484078153)

[Table 1: PID –AI/I and PID +AI/I group characteristics 10](#_Toc484078154)

[Figure 1: Flow cytometry plots of T cell subsets 11](#_Toc484078155)

[Figure 2: Flow cytometry plots of regulatory T cells 12](#_Toc484078156)

[Table 2: Comparison of T cell subgroups between groups 13](#_Toc484078157)

[Table 3: Cohort Immunological Investigations 14](#_Toc484078158)

[Figure 3: Distributions of T cell subsets 15](#_Toc484078159)

[Supplementary References 16](#_Toc484078160)

# **Clinical phenotypes**

**P1. *IKBKG* (*NEMO*) c.185G>A:p.(Arg62Gln)**

A 26-year-old male presented with symptoms of acute appendicitis. The patient described recurrent sinopulmonary infections with *Haemophilus influenzae* and chronic diarrhoea since childhood. Imaging revealed splenomegaly and abdominal lymphadenopathy. Histological examination of the appendix and bowel showed a florid intramural lymphocytic infiltrate and colitis with no organisms identified. Isohaemagglutinins were absent and he was hypogammaglobulinaemic with a low IgG 1.6g/l, IgA 0.9g/l and normal IgM 0.7g/l, skewed naïve – memory lymphocyte distributions (Supplementary Table 2). After replacement immunoglobulin, his sinopulmonary infection frequency improved. Stool samples were performed due to persistent diarrhoea, and isolated norovirus repeatedly. He developed autoimmune haemolytic anaemia (AIHA), immune-mediated thrombocytopenia (ITP), and ascities with deranged liver function tests. Liver biopsy showed non-caseating granulomatous inflammation, which was negative for acid-fast bacilli and fungal staining. Genetic analysis identified the variant *IKBKG* (*NEMO*) NM_003639.3 c.185G>A:p.(Arg62Gln) (Polyphen2 0.971(probably damaging), SIFT 0.01 (deleterious), GERP 5.6 ExAC minor allele frequency (MAF) 0.00004). Treatment for his autoimmune complications has required repeated courses of prednisolone (weaning from 1mg/kg daily), and rituximab (4 cycles of 375mg/m^2^). Due to relapsing AIHA, he is now maintained on sirolimus 1mg daily. Norovirus has continued to be isolated from stool samples after immunosuppressive treatment, but his weight has improved and the stool frequency has not increased. At the time of immunological investigation the patient was taking 1mg of sirolimus daily.

**P2. *STAT3* c.1853G>A:p.(Gly618Asp)**

A 33-year-old male presented with *Pneumocystis jiroveci* pneumonia. He had a past infective history stretching back to childhood of recurrent cutaneous staphylococcal boils, fungal nail infections, *Haemophilus influzenae* and *Streptococcus pneumoniae*. He also had previously undergone radical chemoradiotherapy for a testicular cancer and multiple surgical ligament repairs due to hypermobility and dislocations. Immunological assessment showed a raised IgE of 6380 iu/ml, normal IgG, IgA and IgM, but impaired responses to polysaccharide pneumococcal vaccination (Supplementary Table 2). Genetic investigation showed the previously reported pathogenic variant *STAT3* NM_139276.2 c.1853G>A:p.(Gly618Asp) (Polyphen2 0.904(probably damaging), SIFT 0 (deleterious) ExAC MAF 0) resulting in loss-of-function (LOF).^S1^ He has remained well on co-trimoxazole 960mg daily 3 times a week and prophylactic azithromycin 500mg three times a week.

**P3. *STAT3* c.1909G>A:p.(Val637Met)**

A 20-year-old women presented with recurrent pneumonias due to *Haemophilus influenza* and bronchiectasis. She had a history of childhood pneumatoceles that were surgically resected, cutaneous *Staphylococcus aureus* boils, severe eczema, scoliosis and osteoporosis. Genetic analysis identified the variant *STAT3* NM_139276.2 c.1909G>A:p.(Val637Met) (Polyphen2 0.999 (probably damaging), SIFT 0 (deleterious) ExAC MAF 0) which has previous been reported as pathogenic due to *STAT3*_LOF_ causing Hyper IgE Syndrome.^S2^ Subsequently, she has developed an aspergilloma within a new pneumatocele which is managed conservatively with long-term oral voriconazole. She also remains on rotational prophylactic antibiotics with co-trimoxazole, doxycycline, co-amoxiclav, and immunoglobulin replacement.

**P4. *PIK3CD* c.3061G>A:p.(Glu1021Lys)**

A 28-year-old male was referred with bronchiectasis, *Haemophilus influenzae* pneumonia, chronic diarrhoea, chronic mucocutaneous candidasis (CMC) and impaired response to polysaccharide vaccines. Colonoscopy revealed widespread nodular lymphoid hyperplasia throughout the colon. Stool samples were negative for bacterial and viral pathogens. Immunological investigations revealed polyclonal raised IgG and IgM, normal IgA, and CD4^+^ lymphopenia (Supplementary Table 2). IgG subclasses showed an IgG1 of 17.4g/l (raised) IgG2 1.8 (low normal), IgG3 2.5g/l, IgG4 <0.02g/l. The CMC responded to itraconazole. Genetic analysis identified the known pathogenic gain-of-function (GOF) variant *PIK3CD* NM_005026.3 c.3061G>A:p.(Glu1021Lys) (Polyphen2 0.997 (probably damaging), SIFT 0 (deleterious), GERP 3.72, ExAC MAF 0).^S3, S4^ The clinical phenotype of the patient is similar to those reported.^S5^

**P5. *PIK3CD* c.3061G>A:p.(Glu1021Lys)**

The clinical phenotype of P5 has previously been reported.^S6^ Briefly, a 4-year-old boy presented with *Streptococcus pneumoniae* bacteraemia and splenomegaly. Immunological investigations showed CD4^+^ lymphopenia and impaired responses to polysaccharide vaccines. He developed severe IgG direct antiglobulin test (DAT) positive chronic AIHA and small bowel lymphocytic colitis. The GOF variant *PIK3CD* NM_005026.3 c.3061G>A:p.(Glu1021Lys) (Polyphen2 0.997 (probably damaging), SIFT 0 (deleterious), GERP 3.72, ExAC MAF 0) was identified.^S3, S4^ Management of his autoimmune cytopenias has included prednisolone, rituximab, mycophenlate and finally sirolimus. He continues to be managed with sirolimus and immunoglobulin replacement with no further autoimmune relapses or infections. At the time of immunological investigation the patient was taking 2.5mg of sirolimus daily.

**P6. *CTLA4* c.160G>A:p.(Ala54Thr).**

A 49-year-old male presented with chronic non-infectious diarrhoea. Radiological and endoscopic investigations showed basal bronchiectasis and marked interstitial pulmonary fibrosis, and lymphocytic colitis. Open lung biopsy showed lymphoid aggregates and granulation without discrete granulomata. Immunological investigations showed mild hypogammaglobulinaemia and an inverted CD4:8 ratio (Supplementary Table 2). Genetic investigation identified the novel variant *CTLA4* NM_005214.4 c.160G>A:p.(Ala54Thr) (Polyphen2 0.999 (probably damaging), SIFT 0 (deleterious), GERP 4.19, ExAC MAF 0). He began treatment with prednisolone for pulmonary fibrosis and lymphocytic colitis. Immunoglobulin replacement was started due to hypogammaglobulinaemia. The pulmonary fibrosis has continued to progress resulting in pulmonary artery hypertension. He has also suffered repeated pulmonary infections with *Haemophilus influenza, Haemophilus parainfluenzae*, and *Pseudomonas aeruginosa.* During follow up he has developed chronic *Clostridium difficile* infection that has exacerbated his diarrhoea. At the time of immunological investigation the patient was taking 10mg of prednisolone daily.

**P7. *CTLA4* c.118G>A:p.(Val40Met)**

A 30-year-old man presented with multiple autoimmune cytopenias; AIHA, ITP, and autoimmune neutropenia that was treated with prednisolone and rituximab. He was noted to have splenomegaly that did not resolve after remission of the autoimmune cytopenias. He had a background of suffering with psoriasis treated with oral ciclosporin. He suffered disseminated cytomegalovirus (CMV) infection with retinal necrosis whilst on low dose ciclosporin. He was lost to follow-up, but re-presented 10 years later with chronic diarrhoea and recurrent pulmonary infections with *Streptococcus pneumoniae, Staphylococcus aureus, Candida krusei*, and pneumonitis due to Influenzae H1N1. Immunological investigation showed hypogammglobulinaemia and CD4^+^ lymphopenia (Supplementary Table 2). Genetic analysis identified the novel variant *CTLA4* NM_005214.4 c.118G>A:p.(Val40Met) (Polyphen2 0.595 (possibly damaging), SIFT 0.02 (deleterious), GERP 5.28, ExAC MAF 0). Sirolimus did not control his colitis or psoriasis adequately and he was commenced long term prednisolone, methotrexate, and replacement immunoglobulin. At the time of immunological investigation the patient was taking 10mg of prednisolone daily.

**P8.1 and P8.2 *STAT1* c.821T>A:p.(Arg274Gln)**

P8.1 and P8.2 are two brothers aged 12-years-old (P8.1) and 14-years old (P8.2) who presented in infancy with chronic mucocutaneous candidiasis (CMC). Their mother also has suffered lifelong with CMC and autoantibody negative hypothyroidism. The CMC in all family members responded to itraconazole. Genetic analysis identified the known pathogenic gain of function (GOF) variant *STAT1* NM_139266.2 c.821G>A:p.(Arg274Gln) (Polyphen2 0.88 (possibly damaging) SIFT 0.05 (damaging) ExAC MAF 0).^S7^ P8.1 has suffered with bacterial infections, including periorbital cellulitis due to *Staphylococcus aureus*, pseudomonal otitis media, and *Haemophilus influenza* urinary tract infections. P8.2 has remained free of bacterial infections with prophylactic azithromycin antibiotic. A broader combined immunodeficiency in as has been described in some cases of *STAT1*_GOF_, and there is a slightly lower T cell count and increased γδ T cell percentage observed in P8.2 which does appear to be causing clinical manifestations at this time. ^S8, S9^

**P9.1 *NFKB1* c.904dupT:p.(Ser302Phefs*7)**

A 49-year-old Caucasian male presented with recurrent upper respiratory tract infections predominantly due to *Haemophilus influenzae*, splenomegaly and upper zone lung fibrosis. His immunoglobulins were low with IgG 0.9g/l, IgA <0.07g/l, and IgM 0.1g/l. Lymphocyte subsets showed an increased percentage of effector CD8^+^ T cells and low class switched memory B cells (Supplementary Table 2). At presentation he had a strongly positive IgG DAT prior to immunoglobulin replacement, but with no evidence of decompensated haemolysis. Genetic analysis identified the novel variant *NFKB1* NM_003998.3 c.904dupT:p.(Ser302Phefs*7) (GERP 5.26. ExAC MAF 0.00001). He was managed with immunoglobulin replacement for 2 years before suffering clinical AIHA. Treatment for AIHA required 6 months of prednisolone (1mg/kg/day) with a slow weaning period and 4 cycles of rituximab 375mg/m^2^. Post- rituximab he has become DAT positive again, but without clinical haemolysis at this point.

**P9. *NFKB1* c.904dupT:p.(Ser302Phefs*7)**

P9.2 is the son of P9.1, but presented independently with ITP, AIHA with positive DAT, and autoimmune neutropenia aged 9-years-old. He was noted to have mild splenomegaly on examination, and immunological investigation showed panhypogammaglobulinaemia with an IgG of 3.3g/l, IgA 0.2g/l, IgM 0.2g/l, and absent class-switched memory B cells (Supplementary Table 2). The autoimmune cytopenias were successfully treated with a slow weaning course of prednisolone, starting at 1mg/kg/day, followed by immunoglobulin 2g/kg. There was minimal history of infections with only two courses of oral antibiotics previously given for upper respiratory tract infections with no microbiological organisms confirmed. Imaging and lung function showed no evidence of parenchymal lung disease. There have been no gastrointestinal symptoms reported. Genetic investigation identified the same novel variant seen in P9.1, *NFKB1* NM_003998.3 c.904dupT:p.(Ser302Phefs*7) (GERP 5.26. ExAC MAF 0.00001). Subcutaneous immunoglobulin replacement was commenced and he has remained free of significant infection. His DAT remains positive and platelet count fluctuant (50-150x10^9^/l) which may suggest that in the longer term a steroid sparing immunosuppression may be required.

**P10 *NFKB2* c.2557C>T:p.(Arg853*)**

A 2-year-old female presented with proximal renal tubular acidosis that responded to a long slow weaning course of prednisolone. She suffered with recurrent bacterial pneumonias with *Haemophilus parainfluenzae, Haemophilus influenza,* and *Moraxella catarrhalis* that led to basal bronchiectasis. She was found to have poor antibody responses to polysaccharide vaccination and was commenced on immunoglobulin replacement (Supplementary Table 2). Aged 5-years-old she developed alopecia areata and nail dystrophy. Genetic analysis identified the known pathogenic variant in *NFKB2* NM_001077494.2 c.2557C>T:p.(Arg853*) (ExAC MAF 0).^S10^ She remains well on subcutaneous immunoglobulin replacement.

**P11 *GATA2* c.526A>C:p.(Thr176Pro)**

P11 presented at age 7-years-old with cervical lymphadenopathy, splenomegaly, pulmonary inflammation, AIHA and ITP. Initial investigation showed a lymphopenia of 3.4x10^9^/l, monocytes 0.5x10^9^/l, neutrophils 3.1x10^9^/l, normal T, B, and NK cell numbers but skewed memory profiles within CD4^+^ and CD8^+^ T cells (Supplementary Table 2). Following exclusion of pathogens and malignancy he underwent immunosuppression to control the autoimmune cytopenias consisting of a long slow wean of prednisolone, rituximab and concurrent splenectomy. The immunosuppression rendered him hypogammaglobulinaemic and so immunoglobulin replacement was commenced. He suffered with recurrent sinopulmonary infections due to *Haemophilus influenza.* His lymphocyte count fell and he then remained chronically lymphopenic with CD3^+^ 420 cells/mm^3^, CD4^+^ 280 cells/mm^3^, CD8^+^ 100 cells/mm^3^, CD19^+^ 80 cells/mm^3^, and NK cells 190 cells/mm^3^. Further relapses of autoimmune cytopenias required further courses of prednisolone, rituximab and sirolimus for remission. Genetic analysis identified the novel variant; *GATA2* NM_0326380.4 c.526A>C:p.(Thr176Pro) (Polyphen2 0.979 (probably damaging), SIFT 0.03 (deleterious), GERP 4.01, ExAC MAF 0). The threonine 176 amino acid in GATA2 has been shown to be important for post-translational control of GATA2 during the cell-cycle, therefore this variant is likely to impair GATA2 function.^S11, S12^

P11 contracted *Mycoplasma pneumoniae* infection and suffered a severe further relapse of AIHA that was refractory to red blood cell transfusions, and could not be controlled by methylprednisolone, immunoglobulin or rituximab, and caused multi-organ in combination with disseminated infection that resulting in death.

**P12 *STXBP2* c.1247-1G>C homozygous**

A 5-year-old male presented with Epstein-Barr virus (EBV) triggered haemophagocytic lymphohistiocytosis (HLH). He was treated with rituximab, prednisolone and immunoglobulin due to persistent EBV viraemia and deranged liver function with hepatosplenomegaly. His neutropenia was partially steroid responsive and gradually improved. Aged 14-years-old he represented with a recurrent of EBV viraemia and acute hepatitis. A liver biopsy showed evidence of autoimmune sclerosing cholangitis and ursodeoxycholic acid with prednisolone was commenced. Immunoglobulin replacement was started due to concerns regarding persistently low B cells following rituximab and a currently undefined primary immunodeficiency. Genetic analysis identified the previously reported canonical splice site pathogenic variant *STXBP2* NM_006949 c.1247-1G>C (ExAC MAF 0.000676).^S13-S15^ *STXBP2* variants have been reported to compass a clinical phenotype that extends beyond HLH to include low B cells, autoimmunity, autoimmune hepatitis and hypogammaglobulinaemia.^S16, S17^ On-going management consists of immunoglobulin, prophylactic co-trimoxazole and ursodeoxycholic acid and EBV viraemia monitoring. He has suffered with intermittent cutaneous HSV1 outbreaks that have required courses of aciclovir.

**P13 *CD40LG* c.421G>C:p.(Ala141Pro)**

A 1-year-old boy presented with tonsillitis and difficulty swallowing. He was commenced on oral penicillin but did not improve. Due to increasing tonsil size with necrosis he was admitted to intensive care. Swabs and tonsil biopsies grew *Pseudomonas aeruginosa*. Immunological investigations showed an absent IgG, IgA and an elevated IgM at 12.6g/l (Supplementary Table 2). His neutrophil count was low at 0.1x10^9^/l and GSCF was given during the acute infection. Flow cytometry showed absent expression of CD40LG on activated T cells. A diagnosis of X-linked Hyper IgM Syndrome was made and co-trimoxazole prophylaxis and immunoglobulin replacement were commenced. Genetic analysis identified the novel variant *CD40LG* NM_000074.2 c.421G>C:p.(Ala141Pro) (Polyphen2 0.991 (probably damaging), SIFT 0.15 (tolerated), ExAC MAF 0).

Now aged 6-years-old, on immunoglobulin replacement and co-trimoxazole he has remained well. His neutrophil count has normalised and remained between 1.3-5.1x10^9^/l. IgG has been maintained between 8.5 – 12.5g/l and his IgM has fallen in the normal range remaining 1.2 – 1.7 g/l.

**P14 *TAZ* c.658A>G:p.Lys220Glu**

A 33-year-old man was referred following a *Streptococcus pneumoniae* bacteraemia. He had a background of congenital cyanotic heart disease with Tetralogy of Fallot that had been surgically corrected during infancy. He had since suffered with numerous invasive bacterial infections including *Streptococcus pneumoniae* pneumonia, *Streptococcus agalactiae* endocarditis, and *Neisseria meningitidis* meningitis. Immunological investigations showed fluctuant mild neutropenia of 1 - 1.9x10^9^/L, a chronic lymphopenia with CD3^+^ CD4^+^ = 110 cells/mm^3^, normal immunoglobulins, normal complement function, and a normal neutrophil dihydrorhodamine test. HIV antibody and P24 antigen was negative and he is EBV and CMV naïve. Genetic analysis identified the novel variant *TAZ* NM_181313.2 c.658A>G:p.Lys220Glu (PolyPhen-2 0.94 (probably damaging), SIFT 0 (deleterious), GERP 5.33, ExAC MAF 0) which is likely pathogenic.^S18^ He was commenced on co-trimoxazole 960mg three times a week and has suffered no further significant bacterial infections. Chronic fatigue and lethargy have continued to be on going limitations on quality of life.

# **Table 1: PID –AI/I and PID +AI/I group characteristics**

|  | **PID -AI/I** | **PID +AI/I** |
| --- | --- | --- |
| **Number** | 7 | 9 |
| **Mean Age years (+ SEM)** | 23.1 (5.0) | 26.4 (6.3) |
| **Male : Female** | 5 : 2 | 8 : 1 |
| **Genes** | *STAT3, PIK3CD, STAT1, CD40LG, TAZ* | *IKBKG, CTLA4, NFKB1, NFKB2, GATA2, STXBP2* |

Details of the PID without autoimmunity/inflammation (PID –AI/I) and PID with autoimmunity/inflammation (PID +AI/I) groups. The groups did not show a significant difference in mean and standard error of the mean (SEM) for age (*p* =0.69 unpaired *t* test).

# **Figure 1: Flow cytometry plots of T cell subsets**


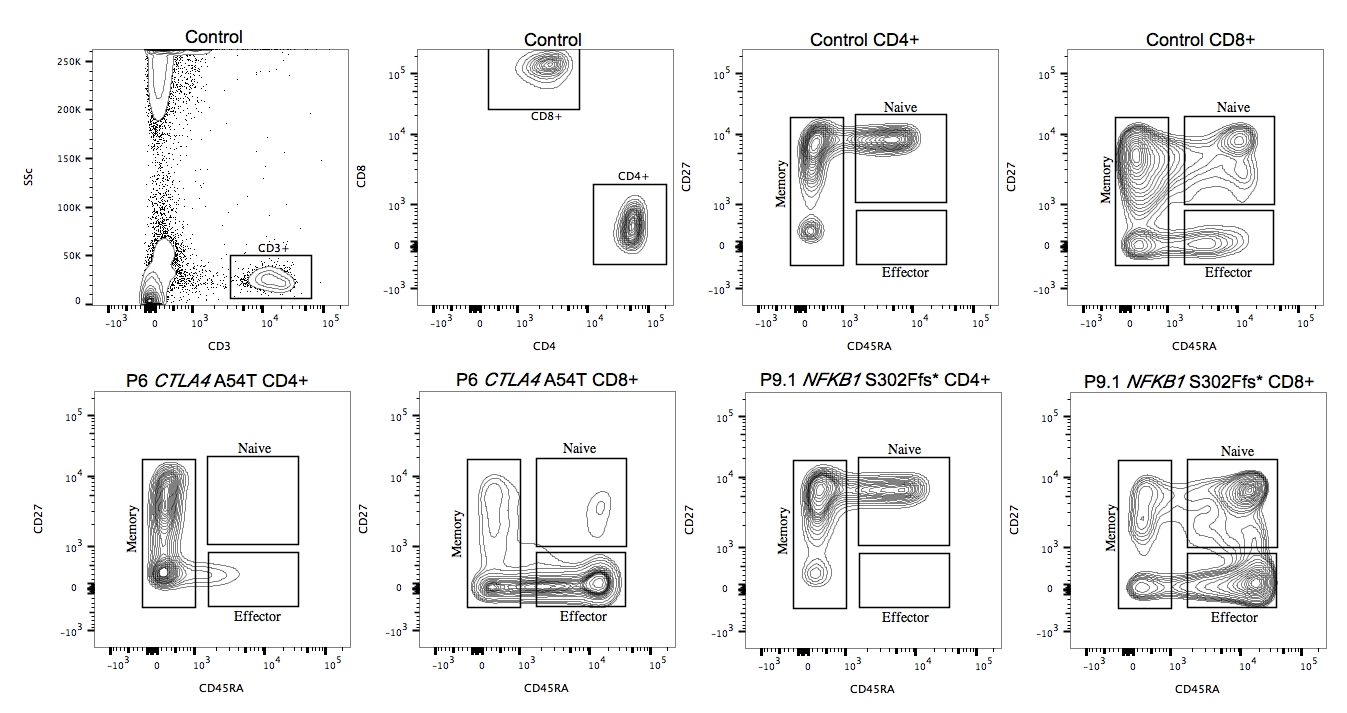


Flow cytometry plots illustrating gating strategy in a control for T cell subsets. Examples of abnormal T cell subset distributions and two cases of PID with AI/I manifestations (P6 and P9.1).

# **Figure 2: Flow cytometry plots of regulatory T cells**


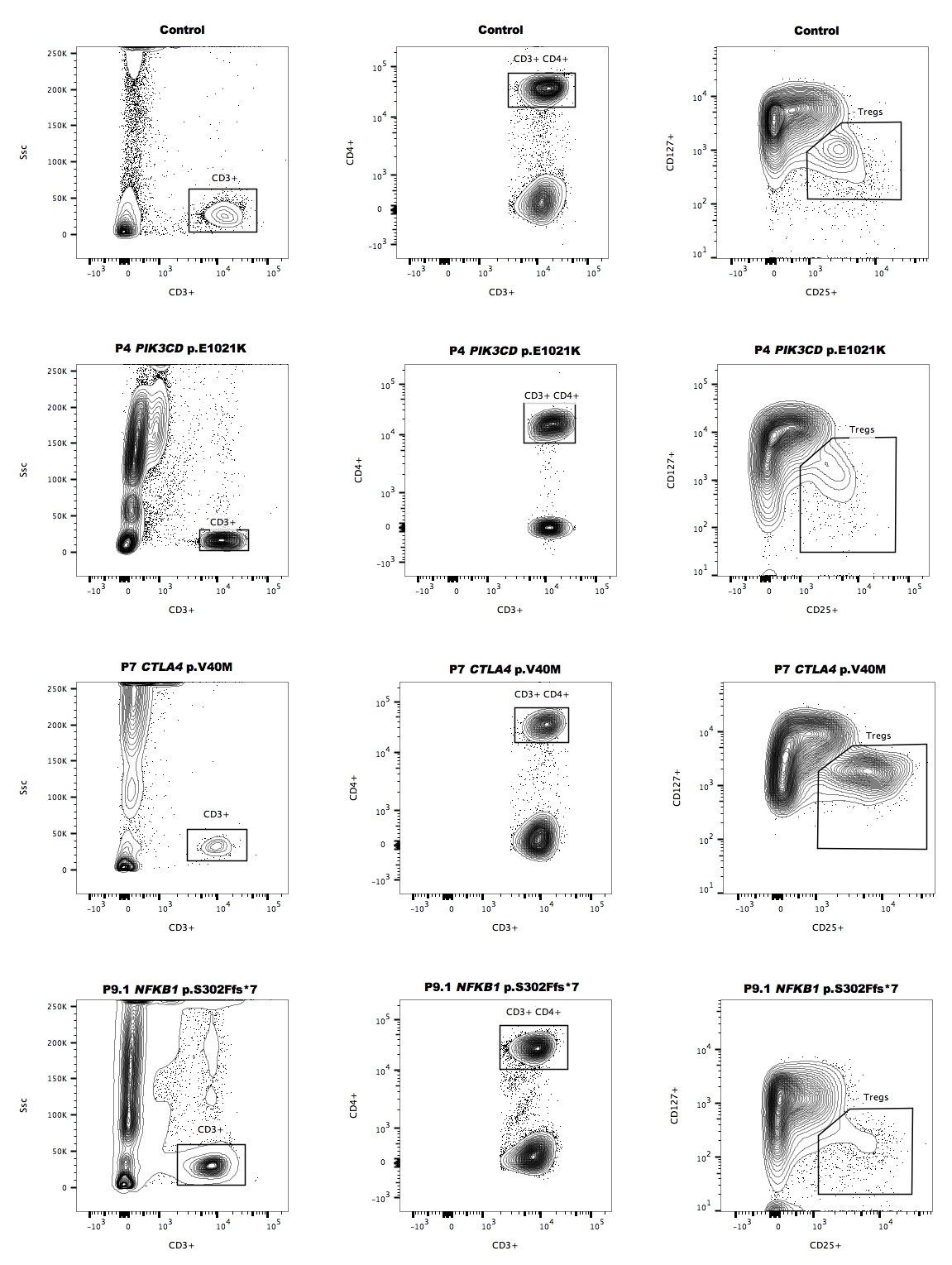


Figure demonstrating the gating for CD3^+^ CD4^+^ CD25^+^ CD127^low^ regulatory Tcells in a control, P4, P7 and P9.1.

# **Table 2: Comparison of T cell subgroups between groups**

|  | **PID –AI/I (Median and interquartile range)** | **PID +AI/I (Median and interquartile range)** | ***p* value** |
| --- | --- | --- | --- |
| **Naïve CD4+** | 40 (21 to 62) | 20 (4.5 to 45.5) | 0.23 |
| **Memory CD4+** | 60 (38 to 63) | 78 (47 to 89.5) | 0.29 |
| **Effector CD4+** | 0 (0 to 6) | 1 (0 to 5.5) | 0.70 |
| **Naïve CD8+** | 33 (18 to 71) | 32 (13.5 to 50) | 0.33 |
| **Memory CD8+** | 41 (28 to 62) | 44 (18 to 56) | 0.62 |
| **Effector CD8+** | 13 (0 to 20) | 21 (12.5 to 51) | 0.067 |
| **Tregs** | 8.2 (6.9 to 9.1) | 5 (3.3 to 6.2) | 0.0079** |

Median and interquartile range of groups across T cell subsets analysed. *p* values calculated by Mann-Whitney U test. Regulatory T cells showed the only significant difference between the two groups. ** = *p* <0.01

# **Table 3: Cohort Immunological Investigations**

|  | **P1** | **P2** | **P3** | **P4** | **P5** | **P6** | **P7** | **P8.1** | **P8.2** | **P9.1** | **P9.2** | **P10** | **P11** | **P12** | **P13** | **P14** |
| --- | --- | --- | --- | --- | --- | --- | --- | --- | --- | --- | --- | --- | --- | --- | --- | --- |
| **Genetic diagnosis** | *IKBKG*  R62Q | *STAT3*  G618D | *STAT3*  V637M | *PIK3CD*  E1021K | *PIK3CD*  E1021K | *CTLA4*  A54T | *CTLA4*  V40M | *STAT1*  R274Q | *STAT1*  R274Q | *NFKB1*  S302Ffs*7 | *NFKB1*  S302Ffs*7 | *NFKB2*  R853* | *GATA2*  T176P | *STXBP2*  c.1247-1G>C | *CD40LG*  A141P | *TAZ*  K220E |
| **Lymphocytes (cells/mm^3^)** | 1600 | 1800 | 2000 | 1400 | 1000 | 1700 | 600↓ | 1200 | 1500 | 1900 | 2100 | 2700 | 3400 | 2200 | 2400 | 400↓ |
| **CD3+ (cells/mm^3^)** | 1250 | 1380 | 1180 | 570↓ | 650↓ | 1500 | 580↓ | 780↓ | 1310 | 1470 | 1160 | 3200 | 1020 | 1910 | 1600 | 180↓ |
| **CD3+CD4+** | 860 | 820 | 850 | 190↓ | 340↓ | 440↓ | 180↓ | 340↓ | 780 | 700 | 640 | 2510 | 580 | 750 | 1030 | 110↓ |
| **Naive** CD4+ CD27+ CD45RA+ (%) | 7↓ | 39 | 60 | 21 | 42 | 2↓ | 20 | 40 | 76 | 49 | 40 | 87 | 2↓ | 10↓ | 62 | 17 |
| **Memory** CD4+ CD27+/-CD45RA- (%) | 90 | 61 | 38 | 73 | 58 | 89 | 78 | 60 | 24 | 36 | 59 | 13 | 95 | 89 | 38 | 63 |
| **Effector** CD4+ CD27-CD45RA+ (%) | 3 | 0 | 2 | 6 | 0 | 9↑ | 2 | 0 | 0 | 15↑ | 1 | 0 | 0 | 1 | 0 | 20↑ |
| **T regulatory cell**  (% of CD4+) | 2.9↓ | 6.8 | 6.9 | 7.3 | 6 | 3.4↓ | 23.3↑ | 8.3 | 8.2 | 5 | 3.1↓ | 3.7↓ | 5.3 | 6.4 | 15.4↑ | 9.1 |
| **CD3+CD8+** | 320 | 500 | 290 | 340 | 130↓ | 1020 | 370 | 280↓ | 470 | 640 | 410 | 650 | 340 | 1140 | 350 | 60↓ |
| **Naive** CD8+ CD27+ CD45RA+ | 26 | 27 | 33 | 15 | 32 | 4 | 12 | 71 | 92 | 32 | 68 | 91 | 32 | 15 | 68 | 18 |
| **Memory** CD8+ CD27+/- CD45RA- (%) | 22 | 54 | 41 | 72 | 48 | 44 | 57 | 28 | 8 | 18 | 18 | 8 | 57 | 55 | 32 | 62 |
| **Effector** CD8+ CD27- CD45RA+ (%) | 52 | 19 | 26 | 13 | 20 | 52 | 31 | 1 | 0 | 50 | 14 | 1 | 11 | 30 | 0 | 20 |
| **T cell proliferation** (PHA stimulation) | Impaired | NA | NA | Impaired | NA | NA | NA | NA | NA | NA | NA | Normal | Impaired | NA | NA | Impaired |
| **γδ TCR**  (CD3 %) | 0↓ | 5 | 4 | 4 | 3 | 0 | 5 | 14 | 3 | 8 | 10 | 2 | 0 | 0 | 13 | 4 |
|  | **P1** | **P2** | **P3** | **P4** | **P5** | **P6** | **P7** | **P8.1** | **P8.2** | **P9.1** | **P9.2** | **P10** | **P11** | **P12** | **P13** | **P14** |
| **B cells** CD19+ (cells/mm^3^) | 60↓ | 300 | 180 | 40↓ | 90↓ | 20↓ | 0↓^†^ | 220 | 150 | 140 | 360 | 240 | 510 | 150 | 460 | 70 |
| CD19+ CD27- IgM+ (%) | 100 | 90 | 87 | 91 | 76 | 54 | 0^†^ | 93 | 93 | 56 | 99 | 97 | 80 | 90 | 97 | 96 |
| CD19+ CD27+ IgM+ (%) | 0 | 8 | 9 | 7 | 20 | 1 | 0^†^ | 4 | 6 | 44 | 1 | 3 | 20 | 8 | 3 | 3 |
| CD19+ CD27+ IgM- (%) | 0 | 2 | 4 | 2 | 4 | 4 | 0^†^ | 3 | 0 | 1 | 0 | 0 | 0 | 2 | 0 | 1 |
| **NK cells**  CD16/56+ (cells/mm^3^) | 320 | 120 | 180 | 70 | 50↓ | 120 | 20 | 160 | 30 | 270 | 480 | 230 | 780 | 110 | 270 | 130 |
| **IgG (g/l)** | 1.5↓ | 13.4 | 10.2 | 25.7↑ | 9.4 | 4.3↓ | 3.7↓ | 15.2 | 9.6 | 0.9↓ | 3.3↓ | 5.8↓ | 12.3 | 19.3↑ | <0.3*↓ | 15.6 |
| **IgA (g/l)** | 0.01↓ | 0.76 | 1.4 | 1.87 | 0.5↓ | 0.1↓ | 1.2 | 1.12 | 0.55 | <0.07 | 0.2↓ | 2.12 | 2.2 | 0.8 | <0.07*↓ | 2.8 |
| **IgM (g/l)** | 0.7 | 0.7 | 1.5 | 3.5↑ | 0.8 | 0.1 | 0.5 | 0.7 | 0.4 | 0.1↓ | 0.2↓ | 0.6 | 1.3 | 1.3 | 12.6*↑ | 1.2 |
| **IgE(iu/ml)** | <5 | 6380↑ | 3470↑ | 17 | 101 | <5 | <5 | 7.7 | 35 | <5 | 10 | <5 | <5 | 217 | NA | NA |
| **DAT** | Positive | Negative | Negative | Negative | Positive | Negative | Positive | ND | ND | Positive | Positive | NA | Positive | Negative | NA | NA |

Clinical phenotype including; infections, autoimmune/inflammatory manifestations, and immunology laboratory data of participants. DAT = direct anti-globulin test. ^†^Results 5 years post rituximab. *Immunoglobulins performed at 1yr of age prior to commencing immunoglobulin replacement. Normal range taken from Schatorje et al., 2011 ^19^ NA = not available

# **Figure 3: Distributions of T cell subsets**

| 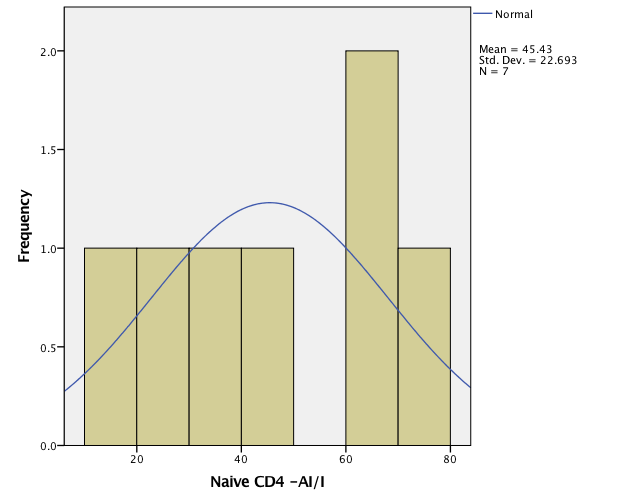 | 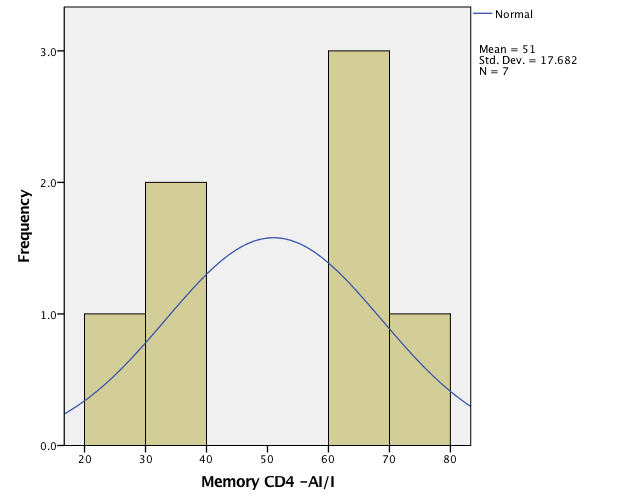 | 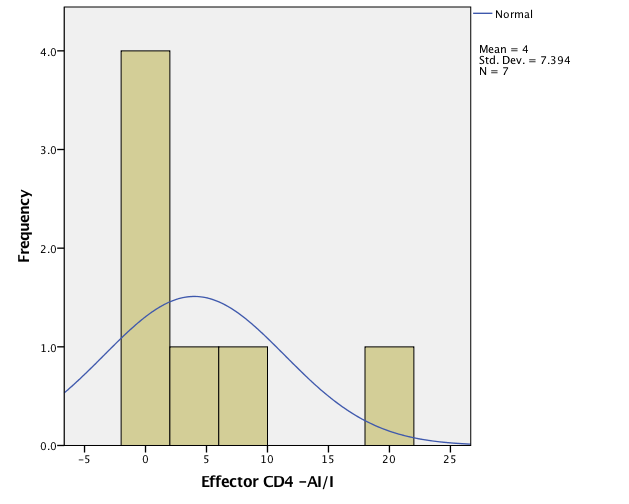 | 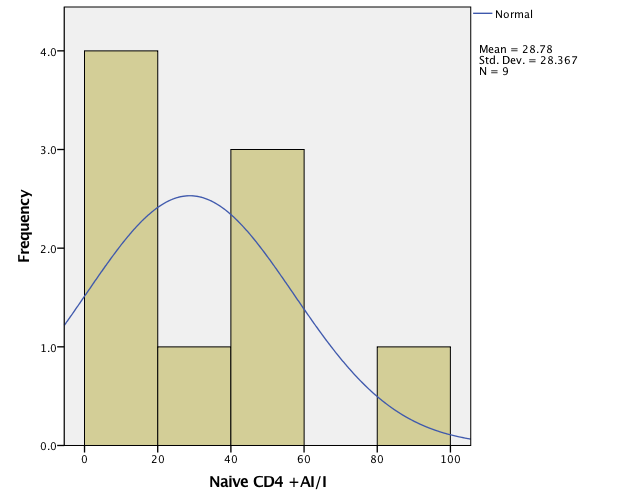 | 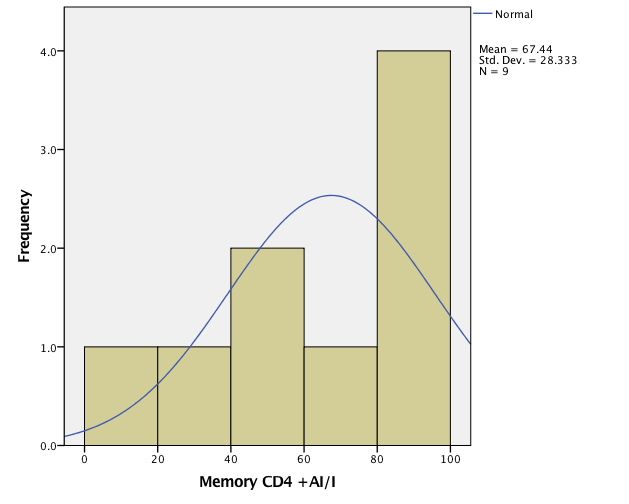 | 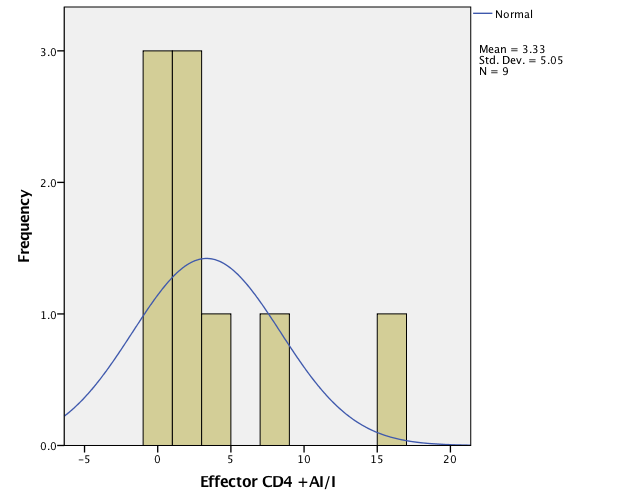 |
| --- | --- | --- | --- | --- | --- |
| 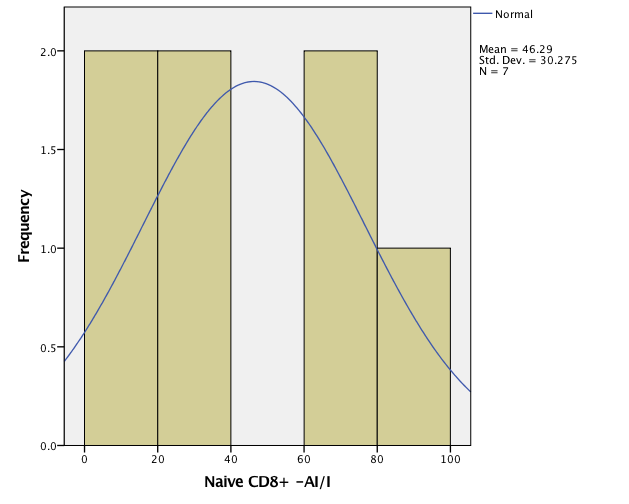 | 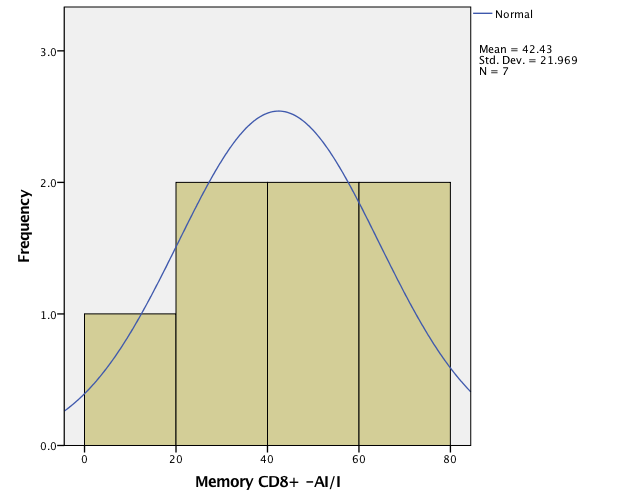 | 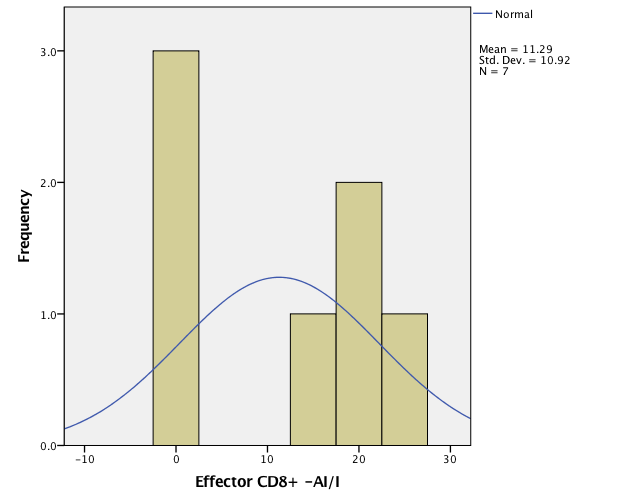 | 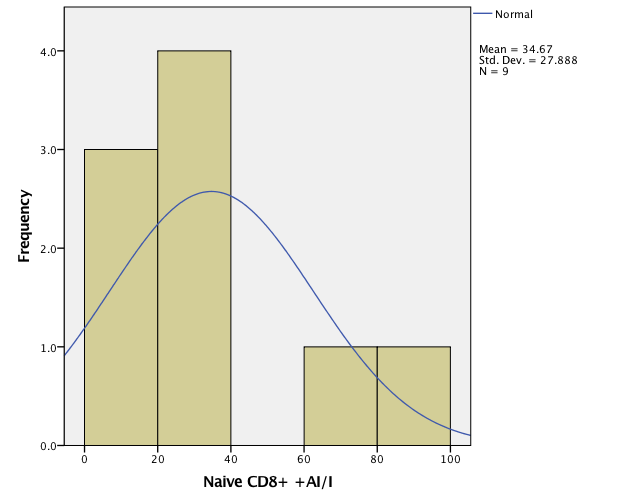 | 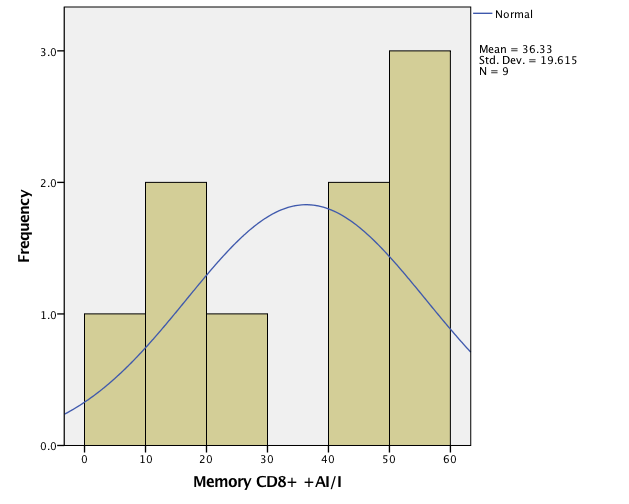 | 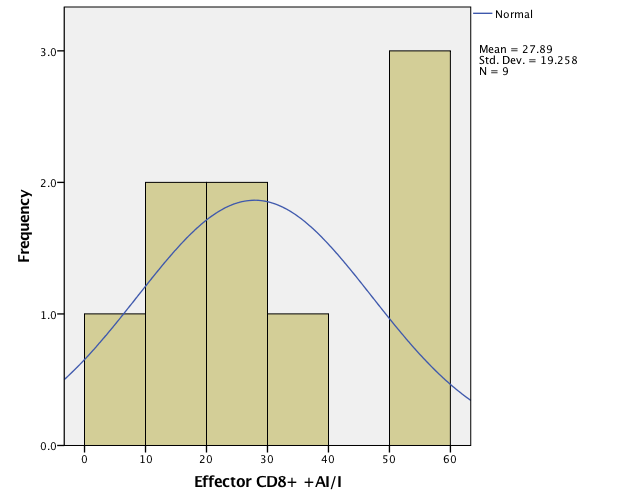 |
|  |  | 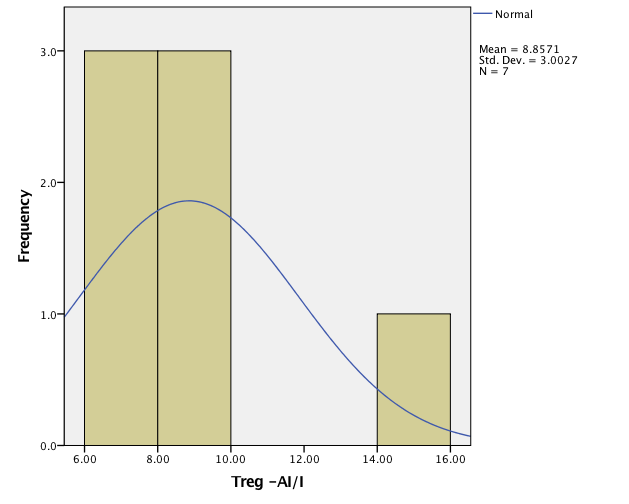 | 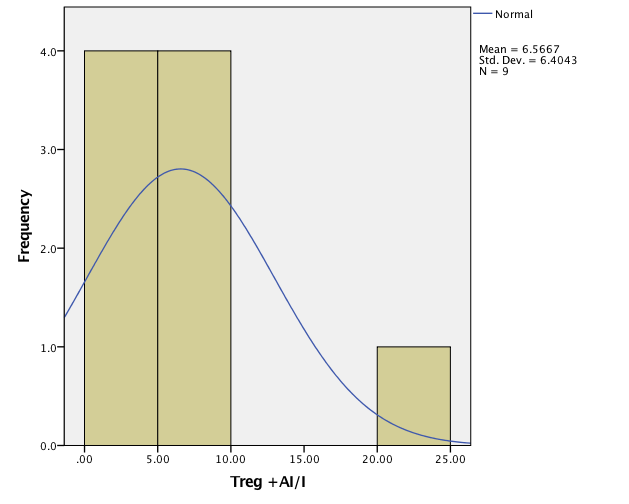 |  |  |

Histograms show non-Gaussian distributions for many of the T cell subsets within the PID cohort. Non-parametric Mann Whitney U test was used in statistical analysis of groups PID –AI/I and PID +AI/I.

# **Supplementary References**

S1. Freeman AF, Renner ED, Henderson C, Langenbeck A, Olivier KN, Hsu AP, et al. Lung parenchyma surgery in autosomal dominant hyper-IgE syndrome. *J Clin Immunol* 2013; **33**: 896-902.

S2. Holland SM, DeLeo FR, Elloumi HZ, Hsu AP, Uzel G, Brodsky N, et al. STAT3 mutations in the hyper-IgE syndrome. *N Engl J Med* 2007; **357**: 1608-1619.

S3. Angulo I, Vadas O, Garçon F, Banham-Hall E, Plagnol V, Leahy TR, et al. Phosphoinositide 3-kinase δ gene mutation predisposes to respiratory infection and airway damage. *Science* 2013; **342**: 866-871.

S4. Lucas CL, Kuehn HS, Zhao F, Niemela JE, Deenick EK, Palendira U, et al. Dominant-activating germline mutations in the gene encoding the PI(3)K catalytic subunit p110δ result in T cell senescence and human immunodeficiency. *Nat Immunol* 2014; **15**: 88-97.

S5. Coulter TI, Chandra A, Bacon CM, Babar J, Curtis J, Screaton N, et al. Clinical spectrum and features of activated phosphoinositide 3-kinase δ syndrome: A large patient cohort study. *J Allergy Clin Immunol* 2016; **139**: 597-606

S6. Rae W, Ramakrishnan KA, Gao Y, Ashton-Key M, Pengelly RJ, Patel SV, et al. Precision treatment with sirolimus in a case of activated phosphoinositide 3-kinase δ syndrome. *Clin Immunol* 2016; **171**: 38-40.

S7. Takezaki S, Yamada M, Kato M, Park MJ, Maruyama K, Yamazaki Y, et al. Chronic mucocutaneous candidiasis caused by a gain-of-function mutation in the STAT1 DNA-binding domain. *J Immunol* 2012; **189**: 1521-1526.

S8. Baris S, Alroqi F, Kiykim A, Karakoc-Aydiner E, Ogulur I, Ozen A, et al. Severe Early-Onset Combined Immunodeficiency due to Heterozygous Gain-of-Function Mutations in STAT1. *J Clin Immunol* 2016; **36**: 641-648.

S9. Sharfe N, Nahum A, Newell A, Dadi H, Ngan B, Pereira SL, et al. Fatal combined immunodeficiency associated with heterozygous mutation in STAT1. *J Allergy Clin Immunol* 2014; **133**: 807-817.

S10. Chen K, Coonrod EM, Kumánovics A, Franks ZF, Durtschi JD, Margraf RL, et al. Germline mutations in NFKB2 implicate the noncanonical NF-κB pathway in the pathogenesis of common variable immunodeficiency*. Am J Hum Genet* 2013; **93**: 812-824.

S11. Nakajima T, Kitagawa K, Ohhata T, Sakai S, Uchida C, Shibata K, et al. Regulation of GATA-binding protein 2 levels via ubiquitin-dependent degradation by Fbw7: involvement of cyclin B-cyclin-dependent kinase 1-mediated phosphorylation of THR176 in GATA-binding protein 2. *J Biol Chem* 2015; **290**: 10368-10381.

S12. Koga S, Yamaguchi N, Abe T, Minegishi M, Tsuchiya S, Yamamoto M, et al. Cell-cycle-dependent oscillation of GATA2 expression in hematopoietic cells. *Blood* 2007; **109**: 4200-4208.

S13. zur Stadt U, Rohr J, Seifert W, Koch F, Grieve S, Pagel J, et al. Familial hemophagocytic lymphohistiocytosis type 5 (FHL-5) is caused by mutations in Munc18-2 and impaired binding to syntaxin 11. *Am J Hum Genet* 2009; **85**: 482-492.

S14. Pagel J, Beutel K, Lehmberg K, Koch F, Maul-Pavicic A, Rohlfs AK, et al. Distinct mutations in STXBP2 are associated with variable clinical presentations in patients with familial hemophagocytic lymphohistiocytosis type 5 (FHL5). *Blood* 2012; **119**: 6016-6024.

S15. Côte M, Ménager MM, Burgess A, Mahlaoui N, Picard C, Schaffner C, et al. Munc18-2 deficiency causes familial hemophagocytic lymphohistiocytosis type 5 and impairs cytotoxic granule exocytosis in patient NK cells. *J Clin Invest* 2009; **119**: 3765-3773.

S16. Maffucci P, Filion CA, Boisson B, Itan Y, Shang L, Casanova JL, et al. Genetic Diagnosis Using Whole Exome Sequencing in Common Variable Immunodeficiency. *Front Immunol* 2016; **7**: 220.

S17. Esmaeilzadeh H, Bemanian MH, Nabavi M, Arshi S, Fallahpour M, Fuchs I, et al. Novel Patient with Late-Onset Familial Hemophagocytic Lymphohistiocytosis with STXBP2 Mutations Presenting with Autoimmune Hepatitis, Neurological Manifestations and Infections Associated with Hypogammaglobulinemia. *J Clin Immunol* 2015; **35**: 22-25.

S18. Richards S, Aziz N, Bale S, Bick D, Das S, Gastier-Foster J, et al. Standards and guidelines for the interpretation of sequence variants: a joint consensus recommendation of the American College of Medical Genetics and Genomics and the Association for Molecular Pathology. *Genet Med* 2015; **17**: 405-424.

S19. Schatorjé EJ, Gemen EF, Driessen GJ, Leuvenink J, van Hout RW, de Vries E. Paediatric reference values for the peripheral T cell compartment. *Scand J Immunol* 2012; **75**: 436-444.
